# Ferulic Acid Prevents Nonalcoholic Fatty Liver Disease by Promoting Fatty Acid Oxidation and Energy Expenditure in C57BL/6 Mice Fed a High-Fat Diet

**DOI:** 10.3390/nu14122530

**Published:** 2022-06-18

**Authors:** Zhixin Luo, Mengqian Li, Qiong Yang, Yuhong Zhang, Fang Liu, Lan Gong, Lin Han, Min Wang

**Affiliations:** 1 College of Food Science and Engineering, Northwest A&F University, Yangling, Xianyang 712100, China; 2018060369@nwafu.edu.cn (Z.L.); limengqian@nwafu.edu.cn (M.L.); qiongyang2019060411@nwafu.edu.cn (Q.Y.); liufang201@nwafu.edu.cn (F.L.); hanlin2019@nwafu.edu.cn (L.H.); 2 Institute of Food Science and Technology, Tibet Academy of Agricultural and Animal Husbandry Sciences, Lhasa 850000, China; zhangyuhong@taaas.org; 3 Microbiome Research Centre, St George and Sutherland Clinical School, University of New South Wales, Sydney, NSW 2052, Australia; l.gong@unsw.edu.au

**Keywords:** ferulic acid, NAFLD, energy expenditure, β-oxidation, ketone body biosynthesis

## Abstract

There is a consensus that ferulic acid (FA), the most prominent phenolic acid in whole grains, displays a protective effect in non-alcoholic fatty liver disease (NAFLD), though its underlying mechanism not fully elucidated. This study aimed to investigate the protective effect of FA on high-fat diet (HFD)-induced NAFLD in mice and its potential mechanism. C57BL/6 mice were divided into the control diet (CON) group, the HFD group, and the treatment (HFD+FA) group, fed with an HFD and FA (100 mg/kg/day) by oral gavage for 12 weeks. Hematoxylin and eosin (H&E) staining and Oil Red O staining were used to evaluate liver tissue pathological changes and lipid accumulation respectively. It was demonstrated that FA supplementation prevented HFD-induced NAFLD, which was evidenced by the decreased accumulation of lipid and hepatic steatosis in the HFD+FA group. Specifically, FA supplementation decreased hepatic triacylglycerol (TG) content by 33.5% (*p* < 0.01). Metabolic cage studies reveal that FA-treated mice have elevated energy expenditure by 11.5% during dark phases. Mechanistically, FA treatment increases the expression of rate-limiting enzymes of fatty acid oxidation and ketone body biosynthesis CPT1A, ACOX1 and HMGCS2, which are the peroxisome proliferator-activated receptors α (PPARα) targets in liver. In conclusion, FA could effectively prevent HFD-induced NAFLD possibly by activating PPARα to increase energy expenditure and decrease the accumulation of triacylglycerol in the liver.

## 1. Introduction

Non-alcoholic fatty liver disease (NAFLD) is the most common liver disease worldwide, with a global prevalence of approximately 25% [1]. It is characterized by excessive triglyceride accumulation in hepatocytes, which can progress to steatohepatitis with different degrees of fibrosis, and represents an indication for liver transplantation with consistently increasing frequency [2]. Recent evidence demonstrated that NAFLD is associated with insulin resistance and type 2 diabetes mellitus [3]. Reputedly, NAFLD is a reversible condition, commonly associated with obesity, and can be reversed with weight loss [4]. However, few effective medicines against NAFLD have been discovered [5]. Lifestyle modifications via diet and exercise remain the highly recommended treatment for NAFLD, but this is hard to maintain in the long term [6]. Increasing evidence shows that a certain number of natural products possess protective or therapeutic bioactivity against NAFLD.

Peroxisome proliferator-activated receptors (PPARs) regulate energy metabolism and are therefore therapeutic targets in NAFLD [3]. Among them, PPARα regulates lipid metabolism by regulating genes involved in fatty acid metabolism, including the β-oxidation and transportation processes of fatty acids. Carnitine palmitoyltransferase 1α (CPT1α) in the liver mitochondrial outer membrane is a rate-limiting fatty acid oxidation enzyme and is responsible for fatty acid transport into mitochondria for further oxidation by converting acyl-CoAs into acylcarnitines [7,8]. In peroxisomes, acyl-CoA oxidase 1 (ACOX1) is the first and a rate-limiting enzyme that catalyzes the desaturation of very-long-chain acyl-CoAs to 2-trans-enoyl-CoAs [9]. Hydroxymethylglutaryl CoA synthase 2 (HMGCS2), generally expressed in liver, is a mitochondrial enzyme that catalyzes the second reaction of ketogenesis from acetyl-CoA [10]; the rate of conversion from acetyl-CoA to these ketone bodies is limited by HMGCS2, which converts acetoacetyl-CoA to 3-hydroxy-3-methylglutaryl-CoA [11]. 

In a state of fasting, metabolic substrates stored in white adipose tissue are released into the circulation as glycerol and free fatty acidsand transported into the liver [11]. The liver then adapts by increasing β-oxidation, which converts fatty acids into acetyl-coA, and by increasing ketogenesis, which converts the resulting acetyl-CoA into ketone bodies. The production of ketone bodies as an alternative energy source is crucial for maintaining energy homeostasis during fasting, as they are used as the main energy source for peripheral tissue [11].

Ferulic acid (FA, 4-hydroxy-3-metoxybenzene acrylic acid) is one of the main natural phenolic acids in cereals including rice [12], rye [13], wheat [14] and barley [15]. Because of its strong hepatoprotective, anti-inflammatory, and antioxidant protective effects, etc. [16], it has been widely applied in the prevention of metabolic diseases, such as cardiovascular diseases [17], and diabetes [18]. Recently, some studies have reported its protective effects against diet-induced NAFLD in a range of rodent models [19,20,21,22], however, the exact mechanism remains unknown.

In the present study, we investigated the mechanism by which FA prevents high-fat-diet-induced NAFLD. This work will help to improve the understanding of the potential mechanism underlying the protective effect of FA on NAFLD.

## 2. Materials and Methods

### 2.1. Chemicals and Reagents

Ferulic acid (FA, purity ≥ 99%) and the β-Hydroxybutyric Acid Content Assay Kit were purchased from Solarbio Science & Technology co., LTD (Beijing, China). Primers (Table 1) used in this study were ordered from Sangon Biotechnology Ltd. (Shanghai, China). The Free Fatty Acid Content Assay Kit was purchased from Beijing Boxbio Science & Technology Co., Ltd. (Beijing, China). Other chemicals and reagents were purchased from Sigma-Aldrich (Darmstadt, Germany) unless specifically stated. 

### 2.2. Animal Experiment

Five-week-old male C57BL/6J mice were obtained from the Experimental Animal Center of Xi’an Jiaotong University (Xi’an, China). The mice were fed a normal chow diet for an acclimatization period of 1 week after their arrival. A schematic diagram of mice treatment is shown in Figure S1. In brief, the mice were randomly divided into 3 groups of 10 mice per group and named CON, HFD and HFD+FA groups. As shown in Table 2, CON, HFD and HFD+FA mice were fed a low-fat diet, a high-fat diet (HFD), and a HFD for 12 weeks, respectively. The low-fat diet (TP23402, TROPHIC Animal Feed High-tech Co. Ltd., Nantong, China) contained 10.0% kcal from fat, 14.1% kcal from protein, and 76.9% kcal from carbohydrate, while the HFD (TP23400) contained 60.0% kcal from fat, 14.1% kcal from protein, and 25.9% kcal from carbohydrate. FA dissolved in sodium carboxymethylcellulose (CMC-Na, 0.3% *w*/*v*) was orally administered to the HFD+FA mice via a gastric tube, while the CON group and the HFD group also received an equal volume of the vehicle, sodium carboxymethylcellulose. Body weight and food intake were recorded daily. At designated experimental endpoints, all mice were anesthetized with isoflurane and cervical dislocation after a 12 h fast. Serum and tissue samples were then collected by either snap-freezing in liquid nitrogen and storing at −80 °C or directly storing in 4% paraformaldehyde for histological analysis. All animal experimental procedures were conducted following the Guide for the Care and Use of Laboratory Animals: Eighth Edition (ISBN-10: 0-309-15396-4). We have complied with all relevant ethical regulations for animal experiments. All the protocols were approved by the Faculty Animal Policy and Welfare Committee of Northwest A & F University, China (Permission ID: 20200528-010), and therefore the animal experiments were carried out in accordance with all animal care regulations, guidelines, and standards.

### 2.3. Dosage Information

The dosage of FA used in this study was calculated based on pilot experiments and the reference at which no toxicity was observed in humans [16,23]. The dose of 100 mg kg^−1^ body weight per day for mice was equivalent to 500 mg FA per day for a mean human weight of 60 kg, according to the formula for dose translation based on body surface area: mouse equivalent dose (mg kg^−1^) = human dose (mg kg^−1^) × (K_m-adult_/K_m-mouse_), Km factor of 3 and 37 for adult mouse (0.02 kg) and adult human (60 kg), respectively [24].

### 2.4. Metabolic Cage Studies 

Mice were individually housed in metabolic cages (Columbus Instruments, Columbus, OH, USA) at room temperature (22 ± 2 °C) and allowed to acclimate for 24 h. After acclimatization, mice were gavaged with FA (100 mg/kg) every morning for 3 days. Oxygen consumption (VO_2_), carbon dioxide production (VCO_2_), and respiratory exchange ratio (RER) were measured every five minutes. The RER was derived from the ratio of VCO_2_ to VO_2_, and energy expenditure was determined as (3.815 + 1.232 × RER) × VO_2_ and expressed as kcal h^−1^. Total body weight was used as the covariate in the analysis.

### 2.5. Metabolic Assay 

At week 12, all mice were fasted overnight prior to the oral glucose tolerance test (OGTT). Mice were gavaged with glucose (2.0 g/(kg of bw)), the glucose levels of tail vein blood samples were measured at 0, 15, 30, 45, 60, 90, and 120 min using a glucose analyzer (Sinocare Inc., Changsha, China). For the insulin tolerance test (ITT), mice were fasted for 6 h and then administered insulin (0.75 units/(kg of bw)) by intraperitoneal injection. The glucose levels of tail vein blood samples were measured at 0, 15, 30, 45, 60, 90, and 120 min after insulin load.

### 2.6. Biochemical Analysis of Serum and Liver Samples 

Serum levels of 3-hydroxybutyric acid were quantified using the β-Hydroxybutyric Acid Content Assay Kit following the manufacturer’s protocol. The hepatic β-hydroxybutyric levels were determined using UPLC-MS/MS. The hepatic TG levels were determined using an automatic biochemical analyzer (Chemray 240, Rayto Life and Analytical Sciences Co., Ltd., Shenzhen, China) and normalized to those of the protein concentrations in the initial homogenate according to the manufacturer’s instructions. 

### 2.7. Liver Histopathology

The murine liver tissues were sequentially fixed with 4% paraformaldehyde, dehydrated with gradient ethanol, embedded in paraffin, and sectioned at 5 μm with a microtome (Leica Microsystems, Wetzlar, Germany). Afterwards, the tissue sections were dried overnight at 37 °C. For H&E staining, the sections were heated at 60 °C for 1 h, deparaffinized, rehydrated, and then stained with hematoxylin and eosin (Solarbio, Beijing, China). Subsequently, the stained sections were dehydrated in gradient ethanol and xylene, sealed with neutral balsam, and air-dried at room temperature. For Oil Red O Staining, the samples were rinsed with PBS and fixed in 10% buffered formalin, then stained with Oil Red O (0.5 g in 100 mL of isopropanol) for 60 min. After discarding the staining solution, isopropanol was added to the samples to elute the retained dyes. After mounting, the sections were visualized and photographed using an optical microscope with camera (Olympus, Tokyo, Japan) at a 100× or 400× magnification. 

### 2.8. Quantitative Real-Time PCR

Total RNA was extracted from the murine liver tissue using AG RNAex Pro Reagent (Accurate Biotechnology Co., Ltd., Changsha China) following the manufacturer’s instructions. For quantitative real-time PCR (qRT-PCR) analysis, the first-strand cDNA was obtained using an Evo M-MLV RT Kit with gDNA Clean for qPCR (Accurate Biotechnology Co., Ltd., Hunan, China), and the mRNAs were quantified using the Bio-Rad CFX96 Touch™ Real Time PCR Detection System (Bio-Rad Laboratories, Inc., Hercules, CA, USA) with β-actin as an endogenous control. The qRT-PCR reaction consisted of 10 μL 2 × SYBR^®^ Premix Ex Taq™ II (CWBIO Bio., China), 0.8 μL specific forward/reverse primer (10 μM), 1 μL cDNA, and ddH2O to a final volume of 20 μL. The quantitative PCR was performed using the following conditions: 95 °C for 5 min, 40 cycles of 95 °C for 5 s, and the optimized annealing temperature for 30 s. There were 6 samples in each group, and the reaction of each sample was performed in duplicate. 

### 2.9. Western Blot Analysis

Liver samples were lysed with RIPA buffer (Beyotime Technology, Shanghai, China) containing 1 mM PMSF (Beyotime Technology, Shanghai, China). Subsequently, the lysate was centrifuged at 12,000× *g* for 5 min. The supernatants were collected and the protein concentrations measured using a BCA Protein Assay Kit (Beyotime Technology, Shanghai, China). Protein lysates were analyzed at a concentration of 40 μg per sample and separated with SDS-PAGE before transferring to the polyvinylidene fluoride (PVDF) membrane (Millipore, Inc., Boston, MA, USA). The membrane was blocked with 5% non-fat milk, followed by incubation with antibodies against β-actin, ACOX1, CPT1α, HMGCS2, or PPARα at 4 °C overnight. The membrane was then incubated with HRP-conjugated secondary antibody (1:5000) at 37 °C for 2 h and labeled in the dark with a chemiluminescence ECL kit (WLA003, Wanleibio, Shenyang, China) according to the manufacturer’s instructions. Finally, the protein bands were visualized with a Bio-Rad Chemidoc (Bio-Rad Laboratories, Inc., Hercules, CA, USA) and the optical density was analyzed with the software ImageJ 1.53. The protein expression was normalized using β-actin (Wanleibio, WL01845, Shenyang, China) as an internal control.

### 2.10. Statistical Analysis 

All numerical values are presented as mean ± SEM. Statistical analyses were conducted using an unpaired *t*-test and one-way analysis of variance (ANOVA), followed by Tukey’s post hoc comparison test. Statistical analyses and figures were performed by Prism v8.2.1 (GraphPad Software Inc., San Diego, CA, USA) or the R package (v3.5.2). * *p* < 0.05; ** *p* < 0.01; *** *p* < 0.001; **** *p* < 0.0001.

## 3. Results

### 3.1. FA Reduces Hepatic Steatosis in HFD-Fed Mice

**Figure 1:**
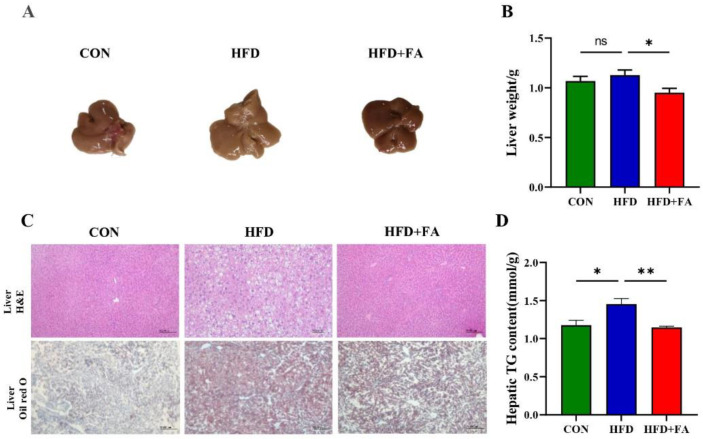
Ferulic acid (FA) reduces hepatic steatosis. (**A**) Representative images of the livers of mice from the three groups CON, HFD, and HFD+FA. (**B**) Liver weight (g) of the mice from the three groups CON, HFD, and HFD+FA (*n* = 10). (**C**) Representative images of (H&E)-stained and Oil Red O-stained liver sections of mice from the three groups, CON, HFD, and HFD+FA at endpoint (12 weeks). Scale bars, 50 µm. (**D**) Liver TG level of mice from the three groups, CON, HFD, and HFD+FA (*n* = 10). Data are shown as mean ± SEM. * *p* < 0.05, ** *p* < 0.01.

Given that NAFLD is a liver manifestation of metabolic syndromes, we first investigate the effect of FA on hepatic lipid accumulation. Male C57BL/6 mice fed an HFD were treated with 100 mg/kg/day of FA or vehicle by gavage for 12 weeks. As shown in Figure 1A, the CON mice fed with the control diet displayed normal liver histology. However, the HFD mice exhibited classical steatosis compared with that of the normal control mice. FA supplementation significantly reduced the liver size (Figure 1A) and weight (Figure 1B) compared with the HFD group. Furthermore, liver H&E staining and Oil Red O staining showed that FA supplementation reduced HFD-induced hepatic steatosis, as evidenced by a reduction in vacuoles and Oil Red O-stained area in the liver (Figure 1C). Statistical results show that FA supplementation decreased hepatic TG content by 33.5% (*p* < 0.01) (Figure 1D).

### 3.2. FA Reduces Expansion of Adipose Tissue and Body Weight Gain in HFD-Fed Mice

**Figure 2:**
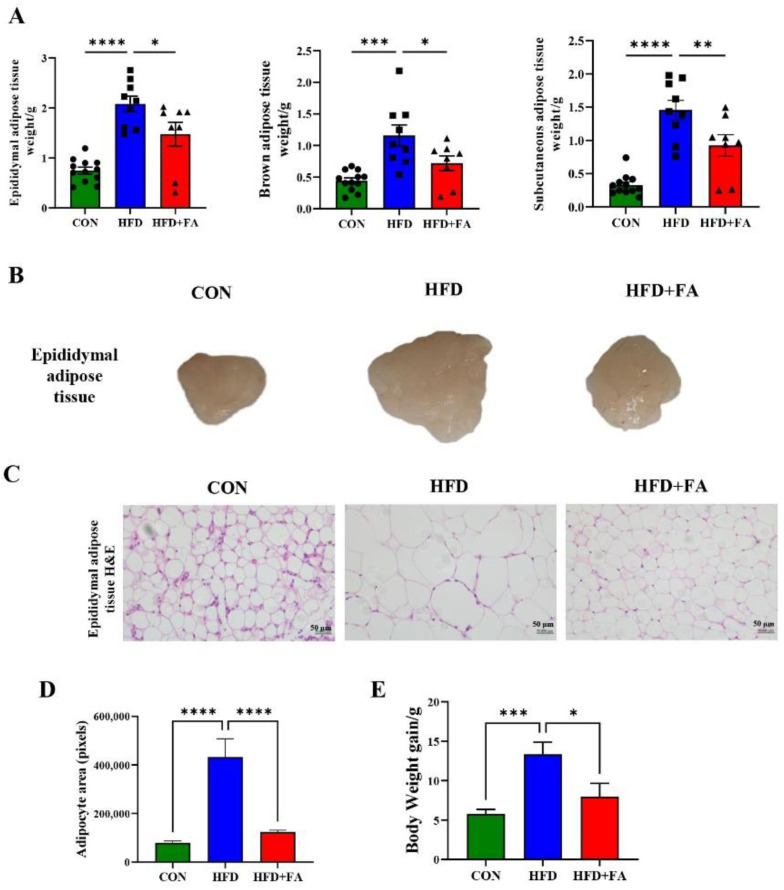
FA reduces expansion of adipose tissue. (**A**) Epididymal, brown and subcutaneous adipose tissue weight (g) in mice from the three groups, CON, HFD, and HFD+FA (*n* = 10). (**B**) Representative images of epididymal adipose tissue in mice from the three groups, CON, HFD, and HFD+FA. (**C**) Representative images of hematoxylin and eosin (H&E)-stained epididymal adipose sections of mice from the three groups, CON, HFD, and HFD+FA at endpoint (12 weeks). Scale bars, 50 µm. (**D**) Distribution of average epididymal adipocyte area of mice from the three groups, CON, HFD, and HFD+FA. (**E**) Body weight gain of mice from the three groups, CON, HFD, and HFD+FA at endpoint. Data are shown as mean ± SEM. * *p* < 0.05, ** *p* < 0.01, *** *p* < 0.001, **** *p* < 0.0001.

Adipose tissue is the primary site of storage for excess energy as triglyceride [25]. We next investigated the effect of FA on the weight of epididymal, brown and subcutaneous adipose tissue. As shown in Figure 2A, FA supplementation significantly reduced the weight of epididymal, brown and subcutaneous adipose, as well as the size of epididymal adipose compared with the HFD group (Figure 2B). Microscopically, the size of epididymal adipocytes was markedly increased in the HFD group compared with the CON group, while FA supplementation significantly reduced the size of epididymal adipocytes (Figure 2C). Distribution of average epididymal adipocyte area showed that the epididymal adipocyte area in HFD+FA group was 71% smaller than that in HFD group (Figure 2D). At the end of the 12-week FA treatment, the body weight gain of the HFD+FA group was significantly reduced by 40.2% (*p* < 0.05) compared with the HFD group (Figure 2E). These results suggest that FA supplementation significantly reduces adipose tissue expansion and body weight gain in HFD fed mice as expected. Interestingly, the energy intake of the HFD+FA mice increased by approximately 0.542 kJ/day/mouse (*p* < 0.01) compared with the HFD group (Figure S2), suggesting that the FA-mediated reduction of expansion in adipose tissue and body weight gain was not due to energy intake decrease.

### 3.3. FA Improves Insulin Sensitivity and Glucose Tolerance

**Figure 3:**
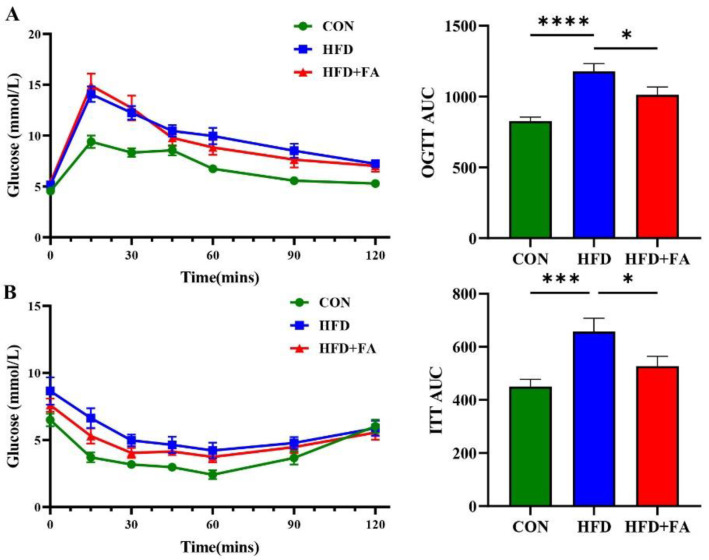
FA improves insulin tolerance and glucose tolerance. (**A**) Oral glucose tolerance test (OGTT) and area under the curve (OGTT-AUC) of mice from three groups, CON, HFD, and HFD+FA. (*n* = 10). (**B**) Insulin tolerance test tests (ITT) and area under the curve (ITT-AUC) of mice from the three groups, CON, HFD, and HFD+FA. (*n* = 10). Data are shown as mean ± SEM. * *p* < 0.05, *** *p* < 0.001, **** *p* < 0.0001.

Since chronic accumulation of fat in the viscera can lead to disturbed glucose metabolism [26], we further assessed the effects of FA supplementation on insulin sensitivity and glucose tolerance using the OGTT and the ITT. These results show reduced area under the curve (AUC) of OGTT (Figure 3A) and ITT (Figure 3B) in HFD+FA mice over the HFD group. Altogether, these results show that FA supplementation improves insulin sensitivity and glucose tolerance in HFD-fed mice.

### 3.4. FA Increased Fatty Acid Oxidation and Ketone Body Biosynthesis in Liver

**Figure 4:**
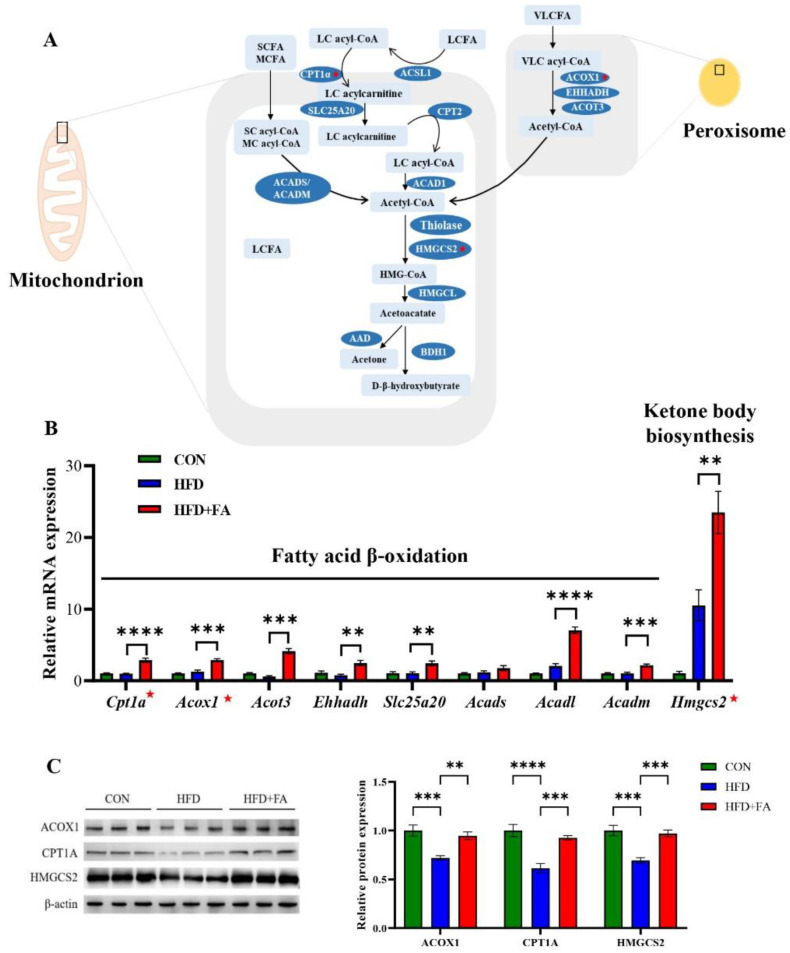
FA increased fatty acid oxidation and ketone body biosynthesis in liver. (**A**) Schematic representation of fatty acid oxidation and ketone body biosynthesis pathways. ★: the rate-limiting enzyme. (**B**) The relative mRNA levels of genes involved in fatty acid oxidation and ketone body biosynthesis in the livers of the CON, HFD and HFD+FA mice (*n* = 6). (**C**) The relative protein levels of CPT1α, ACOX1, and HMGCS2 (*n* = 3). Data are shown as mean ± SEM. ** *p* < 0.01, *** *p* < 0.001, **** *p* < 0.0001.

Given the decrease in hepatic fat accumulation in the HFD+FA group, we examined the expression of hepatic genes involved in fat metabolism, including *Cpt1a*, *Acox1 Acot3*, *Ehhadh*, *Slc25a20*, *Acads*, *Acadl*, *Acadm,* and *Hmgcs2* (Figure 4A). The results show that the mRNA and protein expression of the rate-limiting enzymes of fatty acid oxidation in mitochondria and peroxisomes, CPT1A and ACOX1, respectively, were significantly increased in the HFD+FA group compared with the HFD group (Figure 4B,C). Moreover, we also observe the expression of the rate-limiting enzyme of ketone body biosynthesis, hepatic HMGCS2, was increased in HFD+FA group (Figure 4C) as well as the content of hepatic β-hydroxybutyric acid (Figure S3), indicating that the liver may increase energy output through the conversion of fatty acids to ketone bodies. Unexpectedly, the expression of genes involved in lipolysis and fatty acid oxidation were found to not change in adipose tissue (Figure S4).

### 3.5. FA Activates PPARα in Liver

**Figure 5:**
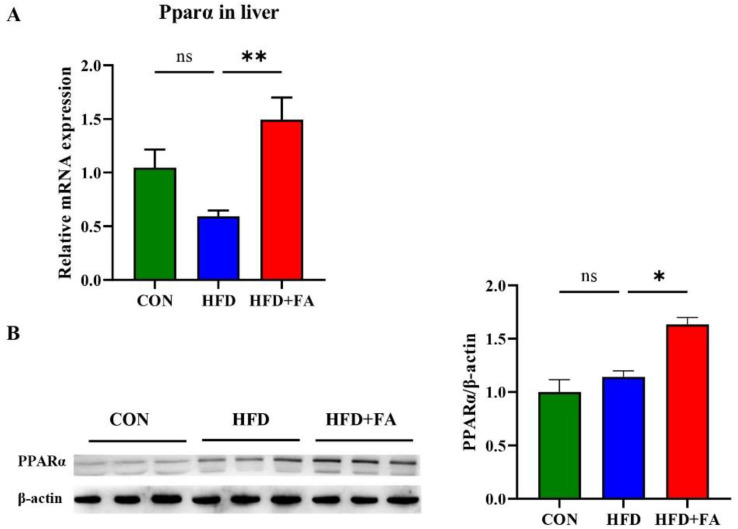
FA activates PPARα in liver. (**A**) The relative mRNA levels of *Ppara* (*n* = 6); (**B**) Western blot analysis of hepatic PPARα protein in the CON, HFD and HFD+FA mice (*n* = 3). Data are shown as mean ± SEM. * *p* < 0.05, ** *p* < 0.01.

Based on the fact that CPT1A, ACOX1, and HMGCS2 are target genes of PPARα, we detected the mRNA and protein expression levels of PPARα in the liver. Compared with the HFD group, the expression of hepatic *Ppara* mRNA and protein in mice from the HFD+FA group increased by 152.2% and 43.0%, respectively (Figure 5A,B). The results show that FA supplementation activates PPARα in liver.

### 3.6. FA Increases Energy Expenditure

**Figure 6:**
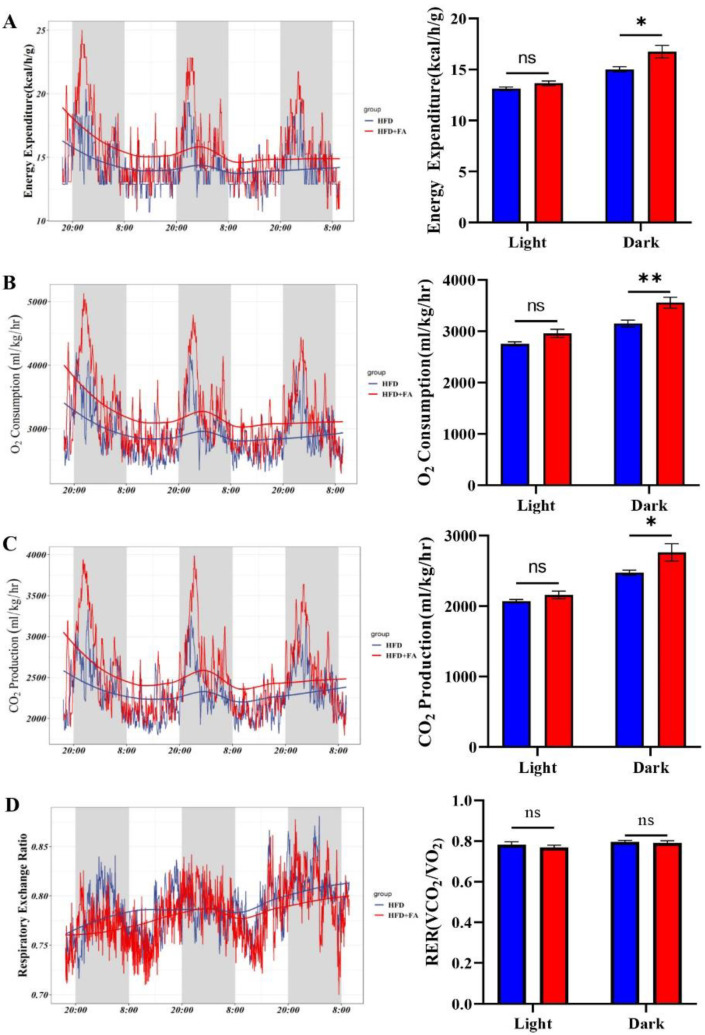
FA increases energy expenditure. (**A**) Energy expenditure; (**B**) oxygen consumption; (**C**) carbon dioxide production; and (**D**) Respiratory exchange ratio (RER) of mice from HFD and HFD+FA groups (*n* = 4). Data are shown as mean ± SEM. * *p* < 0.05, ** *p* < 0.01.

PPARα regulates energy metabolism, so energy expenditure was measured by indirect calorimetry. We found significantly higher energy expenditure, oxygen consumption, and carbon dioxide production (Figure 6A–C) in HFD+FA mice than in HFD mice and this difference was more obvious during dark phases. Compared with the HFD group, energy expenditure, oxygen consumption, and carbon dioxide production of the mice during dark phases in the HFD+FA group increased by 11.5%, 13.0%, and 11.6%, respectively. Meanwhile, RER was slightly lower in HFD+FA mice but not statistically different during both light and dark phases (Figure 6D).

## 4. Discussion

The development and progression of NAFLD is closely associated with an unhealthy dietary pattern [27]. A high energy intake and consumption of specific nutrients directly affects the abnormal accumulation of TG in the liver, a hallmark of NAFLD [2]. Usually, mice receiving HFDs comprising 60% of the total calories, mainly from lard, for 12 weeks is considered as a suitable experimental approach for the development of hepatic steatosis, similar to that found in NAFLD patients [28]. Consistently, the mice fed a 60% HFD for 12 weeks showed marked hepatic steatosis in the study (Figure 1). Furthermore, the mice fed an HFD also exhibited impaired insulin signaling and insulin resistance, since insulin signaling is impaired by the accumulation of lipids in the liver [29]. As expected, FA supplementation decreased hepatic lipid accumulation and improved the hepatic insulin resistance ( Figure 1 and Figure 3), which was consistent with a previous study using *ApoE*^−/−^ mice and rats [30,31].

As a key mediator in regulating energy metabolism, PPARα is a therapeutic target for NAFLD [32]. Fat mass and liver weight are increased in PPARα-deficient mice, [33,34], whereas mice treated with the PPARα agonist, fenofibrate, exhibited decreased body weight and visceral mass [35,36]. Similarly, we demonstrated in this study that FA supplementation significantly increased energy expenditure and the expression of hepatic PPARα, which are consistent with mice treated with fenofibrate ( Figure 4 and Figure 5). Furthermore, some studies have reported that dietary or natural chemicals attenuate metabolic diseases through increasing the expression of PPARα [37,38,39]. Therefore, we propose that FA prevents NAFLD by activating PPARα in HFD-fed mice.

In NAFLD, the rate of fatty acid input (including de novo synthesis with subsequent esterification to triglycerides and fatty acid uptake) typically exceeds the rate of fatty acid output (including secretion of very-low-density lipoprotein and fatty acid oxidation) [40]. Therefore, one of the effective ways to treat NAFLD is to increase fatty acid β-oxidation. Our data demonstrated that genes involved in fatty acid β-oxidation, including *Acot3*, *Ehhadh*, *Slc25a20*, *Acadl*, *Acadm,* and especially the rate-limiting enzymes *Cpt1a* and *Acox1*, are upregulated in the HFD+FA group in comparison with the HFD group (Figure 4), indicating that fatty acid β-oxidation contributes to the preventive effect of FA on NAFLD.

Fatty acid β-oxidation in the liver is accompanied by ketone body biosynthesis [41]. Ketones account for 5% to 20% of the body’s total energy expenditure [42]. The liver converts fatty acids into ketone bodies that then travel to other organs though the blood [42]. As an alternative energy source produced by the fatty acid oxidation in the liver, the ketone bodies are important vectors of energy transport from the liver to extrahepatic tissues, especially during fasting periods when glucose supply is low [43]. In most cases, elevation in ketones is considered favorable because they provide energy efficiently to meet the body’s energy needs [44]. However, elevation in circulating ketone concentration often triggers various pathological complications by activating detrimental pathways that lead to cellular damage [44]. Several harmful effects of ketone body accumulation have been reported in the literature [44]. For example, an increase of ketone concentration in the blood can lead to a drop of blood pH, which can lead to ketoacidosis, a devastating complication [44]. Interestingly, although hepatic ketone body production increased in the HFD+FA group, the concentration of β-hydroxybutyric acid, the main ketone body in the circulation, did not elevate (Figure S5). The reason for this phenomenon remains obscure, but it may be due to the increased use of ketone bodies by extrahepatic tissues, such as the skeletal muscle, heart, and brain.

Of note, the mice treated with FA exhibited stationary RER, indicating that both lipids and carbohydrates used as the energy substrate are elevated. The same phenomenon was also observed in mice treated with a PPARα agonist [45]. The current study has some limitations. First, where the increased energy expenditure is spent in remains unclear. Recent studies have reported that some polyphenols enhance adipose tissue thermogenesis [46,47]. However, this phenomenon was not observed in our study, as constant body temperature and expression of mitochondrial uncoupling protein 1 mRNA was observed (Figure S4). Also, the increase of energy expenditure was only more pronounced during the dark cycle (Figure 4A). The reason for this phenomenon may be that the elimination half-life of FA is short, only 0.2–0.4 h. Furthermore, we did not investigate the effect of FA on fatty acid oxidation and energy expenditure in the context of a low-fat diet. However, previous studies have reported that FA does not affect weight gain in mice fed a low-fat diet [20]. Therefore, FA may have no effect on fatty acid oxidation and energy expenditure in low-fat diet mice. Further investigations are thus needed to explore the precise mechanisms for these effects of FA.

Overall, the present study clearly showed that FA, the most represented phenolic acid in whole grains, was an effective compound to prevent NAFLD development in vivo. The mechanism by which the FA activates PPARα to prevent NAFLD involves the following: Firstly, FA supplementation decreased hepatic TG accumulation via promoting fatty acid β-oxidation and ketone body biosynthesis to increase fatty acid expenditure. Secondly, FA supplementation increased energy expenditure via increased lipids used as the energy substrate. Therefore, these observations may provide a promising candidate for the prevention of NAFLD, and FA can be developed as functional foods or therapeutic medicines.

## Figures and Tables

**Table 1:** Primer sequences used for qRT-PCR.

Gene (Transcript)	Sequences (5′-3′)	Products Length/bp
*Actb* (NM_007393.5)	GGCTCCTAGCACCATGAAG	190
	CGCAGCTCAGTAACAGTCC	
*Cpt1a* (NM_013495.2)	TGACGGCTATGGTGTTTC	124
	CATGGCTTGTCTCAAGTG	
*Ppara* (NM_011144.6)	TCAAGGCCCGGGTCATACTC	164
	CTGGCAGCAGTGGAAGAATC	
*Acox1* (NM_001377522.1)	GCCTGCTGTGTGGGTATGTCATT	150
	GTCATGGGCGGGTGCAT	
*Hmgcs2* (NM_008256.4)	GTGTCCCGTCTAATGGAG	180
	GAGACACCCAGGATTCAC	
*Acot3* (NM_001346701.1)	TGGTCCTGTGTGGGCTCT	107
	TGCCCTCCGAGTGAGTGT	
*Ehhadh* (NM_023737.3)	GGAACCCCCACCCGAAAG	129
	GATGCGACCCAGTGGCTT	
*Fgf21* (NM_020013.4)	GGTGTCAAAGCCTCTAGGTTTC	139
	CATGGGCTTCAGACTGGTACAC	
*Lipe* (NM_010719.5)	TCCTGGAACTAAGTGGACGCAAG	93
	CAGACACACTCCTGCGCATAGAC	
*Pnpla2* (NM_001163689.1)	AAAGATCGAATTCTAGAGCACC	189
	CCACTCCAACAAGCGGA	
*Pygl* (NM_133198.2)	CCGGAGACCGTTCTGTGC	104
	CTCTACGCCCACGATGCC	
*Gck* (NM_010292.5)	CTGGGAGGAACCAACTTCAG	160
	CAGAGATGCACTCAGAGATG	
*Dgat2* (NM_026384.3)	GGCTACGTTGGCTGGTAACT	197
	TCTTCAGGGTGACTGCGTTC	
*Slc2a2* (NM_031197.2)	AGATTGGGCCAGGTCCAATCC	132
	ACTGGAAGCAGAGGGCGATGAC	
*Slc25a20* (NM_020520.5)	GGCTTTGCAGGGATCTTC	142
	AGGTGACTCCTTCTTCTC	
*Acads* (NM_007383.3)	GTGCTGCCATGTTGAAAG	142
	GGCATCTCTGTCACATAC	
*Acadm* (NM_007382.5)	GGAGGCTATGGATTCAAC	119
	ATGTGCTCACGAGCTATG	
*Acadl* (NM_007381.4)	GGTACATGTGGGAGTACC	100
	CTTGCGATCAGCTCTTTC	
*Fasn* (NM_007988.3)	TAAAGCATGACCTCGTGATGAA	230
	GAAGTTCAGTGAGGCGTAGTAG	
*Scd1* (NM_009127.4)	GAACACCCATCCCGAGAGT	176
	TGTAAGAACTGGAGATCTCTTGGA	
*Acaca* (NM_133360.2)	AGTGATGGTGGCCTGCTCTTG	150
	AGCAGACGGTGAGCGCATTA	

**Table 2:** Ingredient composition of experimental diets.

	Group	CON	HFD	HFD+FA
Ingredient (g/kg Diet)		TP23402	TP23400	
Casein		195	195	
Maltodextrin		56	225	
Sucrose		55	89	
Corn starch		479	0	
Soybean Oil		33	33	
Lard		25	301	
Cellulose		69	69	
Mineral Mix, M1020		68	68	
Vitamin Mix, V1010		14	14	
L-Cystine		3	3	
Choline Bitartrate		3	3	
TBHQ		0.067	0.067	
Total		1000	1000	

## Data Availability

Data is contained within the article.

## Supplementary Materials

The following supporting information can be downloaded at: https://www.mdpi.com/article/10.3390/nu14122530/s1, Figure S1: Schematic diagram of mice treatment. Figure S2: Effects of FA supplementation on food intake (A) and energy intake (B) in HFD-fed mice. Figure S3: Effects of FA supplementation on hepatic β-hydroxybutyric acid in HFD-fed mice. Figure S4: The expression of genes involved in lipolysis and fatty acid oxidation in brown adipose tissue. Figure S5: Effects of FA supplementation on serum(A) β-hydroxybutyric acid and free fatty acid (B) concentration in HFD-fed mice.
