# Supplementary material for: Ferulic Acid Prevents Nonalcoholic Fatty Liver Disease by Promoting Fatty Acid Oxidation and Energy Expenditure in C57BL/6 Mice Fed a High-Fat Diet
Source: Nutrients. 2022 Jun 18;14(12):2530. doi: 10.3390/nu14122530 (PMC9230086; doi:10.3390/nu14122530)
Supplement: Supplementary file 1 [file nutrients-14-02530-s001.zip › nutrients-1717937-supplementary.pdf]

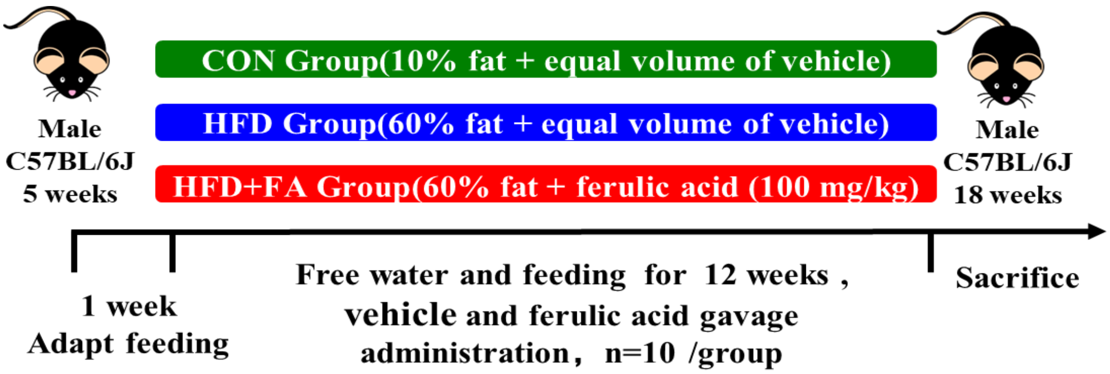

Figure S1: Schematic diagram of mice treatment.

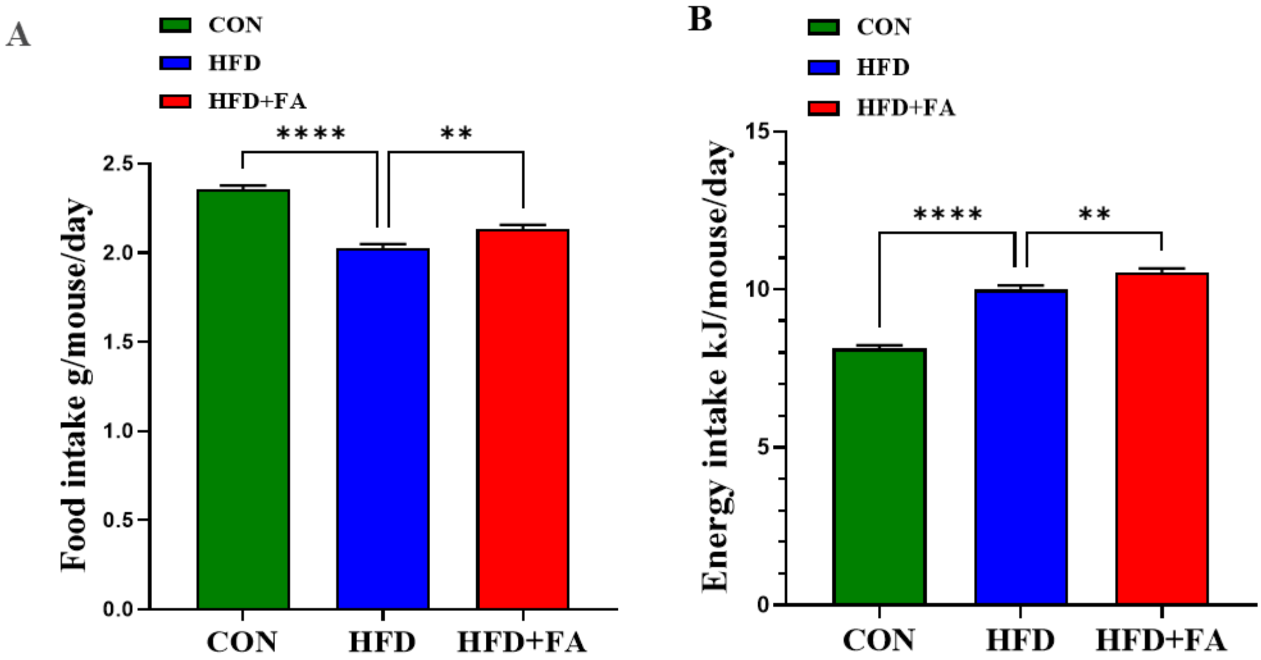

Figure S2: Effects of FA supplementation on food intake (A) and energy intake (B) in HFD-fed mice. Data are represented as mean ± SEM. $^{**}p < 0.01$, $^{****}p < 0.0001$.

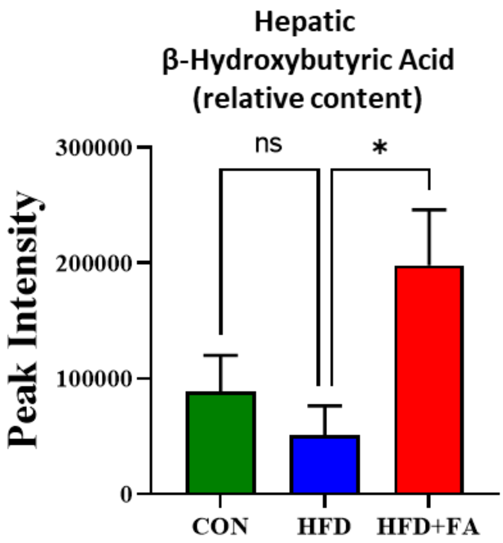

Figure S3: Effects of FA supplementation on hepatic β-hydroxybutyric acid in HFD-fed mice. Data are represented as mean ± SEM. *$p < 0.05$.

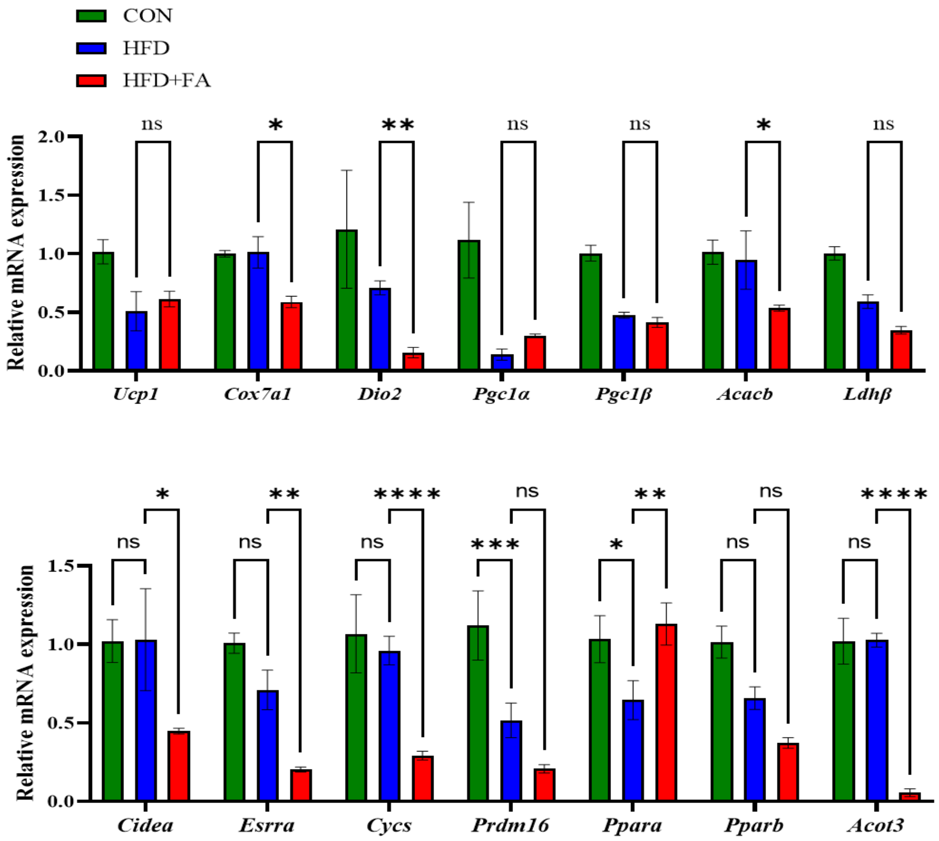

Figure S4: The expression of genes involved in lipolysis and fatty acid oxidation in brown adipose tissue. Data are represented as mean ± SEM. *p < 0.05, **p < 0.01, ***p < 0.001, ****p < 0.0001.

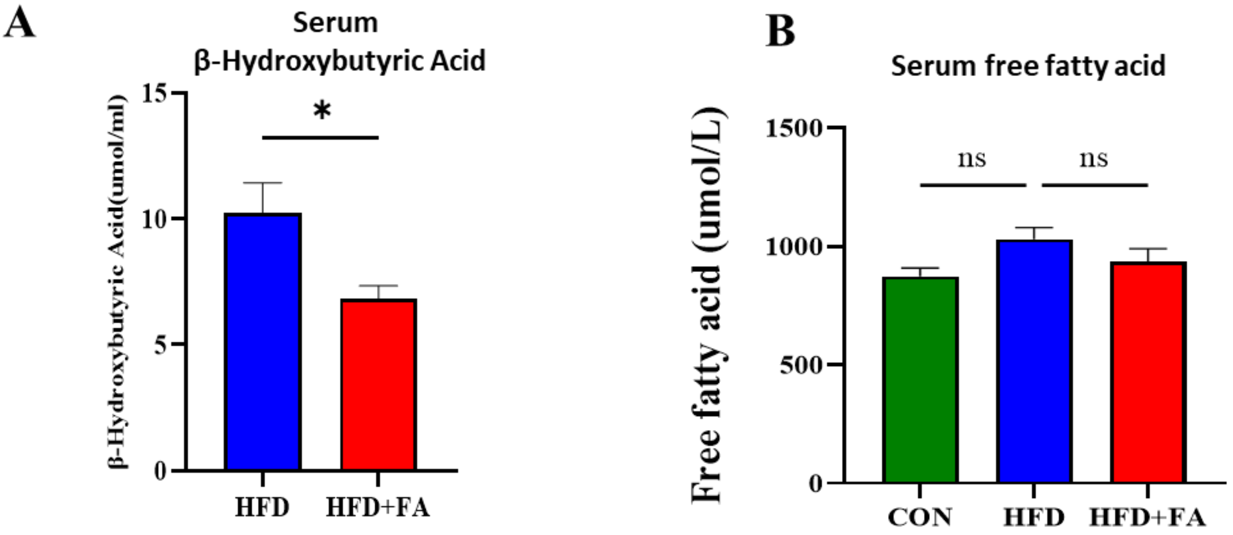

Figure S5: Effects of FA supplementation on serum(A) β-hydroxybutyric acid and free fatty acid (B) concentration in HFD-fed mice. Data are represented as mean ± SEM. *p < 0.05.
